# Acquisition of orthographic forms via spoken complex word training

**DOI:** 10.3758/s13423-022-02185-y

**Published:** 2022-10-17

**Authors:** Elisabeth Beyersmann, Signy Wegener, Jasmine Spencer, Anne Castles

**Affiliations:** 1 School of Psychological Sciences, Australian Hearing Hub, Macquarie University, 16 University Avenue, Sydney, NSW 2109 Australia; 2 Macquarie University Centre for Reading, Macquarie University, Sydney, Australia

**Keywords:** Spoken-word learning, Eye tracking, Morphological processing, Reading acquisition

## Abstract

This study used a novel word-training paradigm to examine the integration of spoken word knowledge when learning to read morphologically complex novel words. Australian primary school children including Grades 3–5 were taught the oral form of a set of novel morphologically complex words (e.g., (/vɪbɪŋ/, /vɪbd/, /vɪbz/), with a second set serving as untrained items. Following oral training, participants saw the printed form of the novel word stems for the first time (e.g., *vib*), embedded in sentences, while their eye movements were monitored. Half of the stems were spelled predictably and half were spelled unpredictably. Reading times were shorter for orally trained stems with predictable than unpredictable spellings and this difference was greater for trained than untrained items. These findings suggest that children were able to form robust orthographic expectations of the embedded morphemic stems during spoken word learning, which may have occurred automatically without any explicit control of the applied mappings, despite still being in the early stages of reading development. Following the sentence reading task, children completed a reading-aloud task where they were exposed to the novel orthographic forms for a second time. The findings are discussed in the context of theories of reading acquisition.

The current study explored whether and how beginning readers benefit from their spoken language knowledge while learning to read. Children are equipped with a wealth of knowledge from their spoken language when they first begin to read, but we still know little about how this knowledge is used and integrated during written language acquisition. It has been previously shown that oral vocabulary knowledge predicts children’s reading acquisition (e.g., Duff et al., 2015; Lee, 2011; Nation & Cocksey, 2009; Nation & Snowling, 2004), and although these results suggest that spoken language exerts an important influence on reading acquisition, it does not explain the mechanisms by which this integration of the information is achieved.

Recently, studies have begun to directly test the cognitive mechanisms involved in the integration of oral vocabulary knowledge in reading (e.g., Johnston et al., 2004; McKague et al., 2008; McKague et al., 2001). A particularly intriguing finding is that spoken-word knowledge can assist with the development of written word knowledge even before the word has been encountered in print. On this view, when a reader has adequate knowledge about phoneme-to-grapheme mappings, they can leverage this to generate expectations about the spellings of words they know orally but have not yet seen in writing. Examinations of this idea, which has been termed the *orthographic skeleton hypothesis* (Wegener et al., 2018), use a novel word-training study design. Following oral training on a set of novel words, participants read trained and untrained novel words for the first time, embedded in sentences. Orally trained items tend to show a large difference in processing efficiency for items with predictable (e.g., the spoken word *‘nesh’* written as *nesh*) compared with unpredictable spellings (e.g., the spoken word *‘coib’* written as *koyb*). This difference between trained items with predictable and unpredictable spellings tends to be larger than that observed for untrained items, suggesting that the influence of prior oral vocabulary knowledge varies as a function of the congruence or incongruence between participants’ orthographic expectancies and the actual orthographic form. This finding has been demonstrated with both developing and skilled readers (Jevtovic et al., 2022; Wegener et al., 2018; Wegener et al., 2020) and may be due to an automatic phoneme-to-grapheme mapping mechanism that operates in the absence of any explicit control (e.g., Huettig et al., 2022), similar to the kind of orthographic influences on spoken-word processing that have been previously evidenced in auditory lexical decision (e.g., Chéreau et al., 2007; Pattamadilok et al., 2007; Perre et al., 2009; Taft et al., 2008; Ziegler et al., 2004; Ziegler et al., 2008).

While this prior work suggests that the integration of spoken vocabulary into reading already occurs before the spoken words are encountered in print, it is less clear how precise the kind of orthographic expectancies are that children set up during oral word exposure. The aim of the current study was to further explore the mechanism by which orthographic skeletons are built during oral word training by asking if the formation of orthographic skeletons is limited to items that provide an exact match with the corresponding spoken form (as has been tested previously) or if orthographic expectancy involves setting up a more complex set of orthographic predictions for not just whole words but also stems embedded in words with multiple morphemes. In skilled readers we have seen this kind of flexibility (Beyersmann, Wegener, et al., 2021) suggesting that experienced readers not only form orthographic skeletons of whole words that provide an exact match between phonology and orthography (e.g., /vɪb/ = *vib*), but also of words embedded in a morphologically complex context (e.g., *vib* in /vɪbɪŋ/). However, it is not clear from these findings if children who are still in the process of learning to read are more limited in their ability and flexibility to develop orthographic expectations from spoken forms. The current study was designed to address this question by examining the acquisition of morphologically complex novel forms in primary school children using an oral word-training paradigm that was carried out over three consecutive days. For children to be able to generate orthographic expectations of embedded morphemic constituents, two critical skills must coincide at the same stage of reading development: the ability to detect morphological structure of spoken words (e.g., Berko, 1958; Bryant & Nunes, 2008; Carlisle, 2000; Deacon & Kirby, 2004) including the identification of embedded stems during oral word training (e.g., Deacon & Bryant, 2006), and the ability to form orthographic skeletons prior to print exposure (e.g., Wegener et al., 2018; Wegener et al., 2020).

A novel word-training paradigm was used within which children were taught the oral forms of novel morphologically complex words (e.g., /vɪbɪŋ/, /vɪbd/, /vɪbz/) consisting of a novel word stem (*vib*) and an existing inflectional affix (“-s”, “-ing”, or “-ed”). Children were trained on three consecutive days without ever seeing the printed forms of the novel complex items. During oral training, participants learned to associate pictures of inventions with the novel complex word forms (e.g., “Professor Parsnip has invented a machine that vibs. The machine is used for drying hats. It is made of plastic and spins.”) but never encountered the embedded morphemic units in isolation. Following training, participants read novel word stems (*vib*), half trained and half untrained, for the first time. The words were embedded in sentences, and eye movements were monitored as participants read silently. Spelling predictability was also manipulated, with half of the stems having a predictable spelling (*vib*) and half an unpredictable spelling (*bype* rather than the predictable counterpart *bipe*). This design allowed us to test if children are proficient at generating an orthographic form of embedded stems (e.g., *vib*) during spoken complex word training (/vɪbɪŋ/). To develop such orthographic expectations, children must use their morphological processing skills (e.g., Beyersmann et al., 2012; Beyersmann, Mousikou, et al., 2021; Quémart et al., 2011; Rastle, 2018) in combination with their ability to map phonemes onto graphemes (e.g., Wegener et al., 2018). We hypothesized that if participants generate an orthographic skeleton of the embedded stems, stems with predictable spellings would have shorter looking times and be less likely to be refixated than would unpredictable spellings, and this difference would be greater for trained than untrained items. We preregistered these predictions along with our method, procedure, and data analysis plans (https://aspredicted.org/3vs5s.pdf).

Following the eye-tracking task, participants completed a preregistered but more exploratory reading-aloud task. Here, participants saw the printed form of the novel stems for the second time and it was less clear if any effects of training or predictability would persist beyond the first orthographic exposure.

## Methods

### Participants

Participants were 53 Australian primary school children (Grades 3–5; 14 female; mean age = 9.36 years, *SD* = 1.01), all English native speakers. Each of the three grade cohorts was randomly split into two groups where the first group was trained on Set 1 (*n* = 28) and the second group on Set 2 (*n* = 25). Children were also assessed on a computerized version of the Castles and Coltheart Reading Test 2 (Castles et al., 2009), including regular words (*bed*), irregular words (*blood*), and nonwords (*norf*), with 40 items each that participants attempted to read aloud in a pseudorandomized, untimed test of increasing difficulty (Table 1).

**Table 1:** Castles and Coltheart Reading Test 2 (CC2)—Mean scores, *z* scores, and percentiles

Grade of participants	Subscale	Mean score	Standardized *z* score	Percentile
3	Regular	32.62	0.18	52.24
	Irregular	20.29	0.17	56.67
	Nonwords	23.95	0.05	48.57
4	Regular	34.46	0.03	51.77
	Irregular	23.08	0.07	52.85
	Nonwords	28.62	−0.01	48.61
5	Regular	35.58	−0.13	46.95
	Irregular	23.42	−0.38	35.68
	Nonwords	29.21	−0.43	33.68

An accuracy score out of 40 for each subscale was obtained and converted into standardized *z* scores and percentiles using Australian normative data (Castles et al., 2009).

### Materials

The materials included 96 morphologically complex novel spoken words, used during training, and 32 novel stems, used during eye tracking (adapted from Beyersmann, Wegener, et al., 2021). Half of the stems were assigned spellings that contained frequent phoneme-to-grapheme mappings and thus were highly predictable from their phonology (e.g., “thog” written as *thog*). The other half were assigned unpredictable spellings (e.g., “feg” written as *phegg*). As a result, predictable and unpredictable items could not be matched for number of letters or bigram frequency, but these were matched across training sets, and all items were matched on number of phonemes. Moreover, despite the variation in spelling predictability, all items were regular for reading in that they could be read aloud correctly using common grapheme–phoneme correspondences. Items were split into two sets, which were matched for consonant/vowel structure. Set 1 served as trained items during oral exposure for half of our participants and Set 2 for the other half (Appendix A). Complex words were created by combining novel stems (“vib”) with three different suffixes (“-ing”, “-ed”, “–s”), resulting in three complex forms for each stem (/vɪbɪŋ/, /vɪbd/, /vɪbz/).

### Procedure

**Fig. 1:**
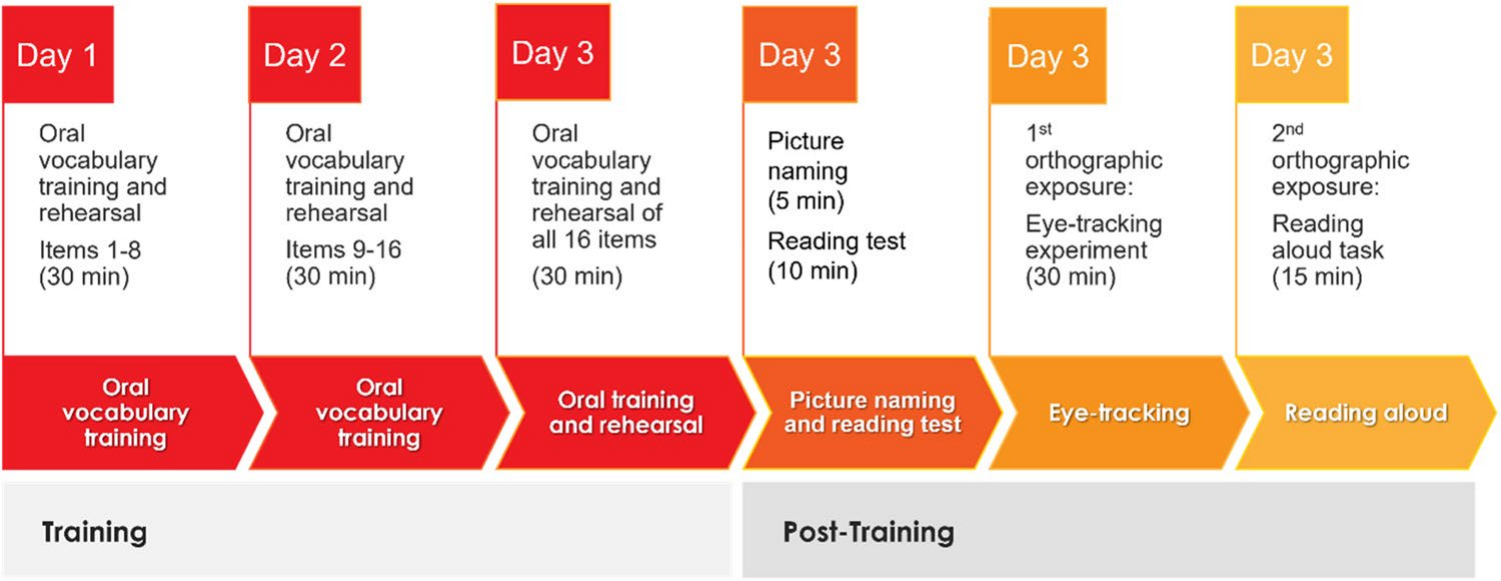
Testing procedure involving three consecutive days of oral vocabulary training at the class level, followed by a range of individual assessment tasks on Day 3

Oral vocabulary training took place over three consecutive days (~30 min/session) to limit the learning load on participants at any one time (Fig. 1). The training was administered in small groups of up to 20 students. Upon completion of the last training session, participants completed individually a range of additional tasks, as described below.

#### Oral vocabulary training

**Fig. 2:**
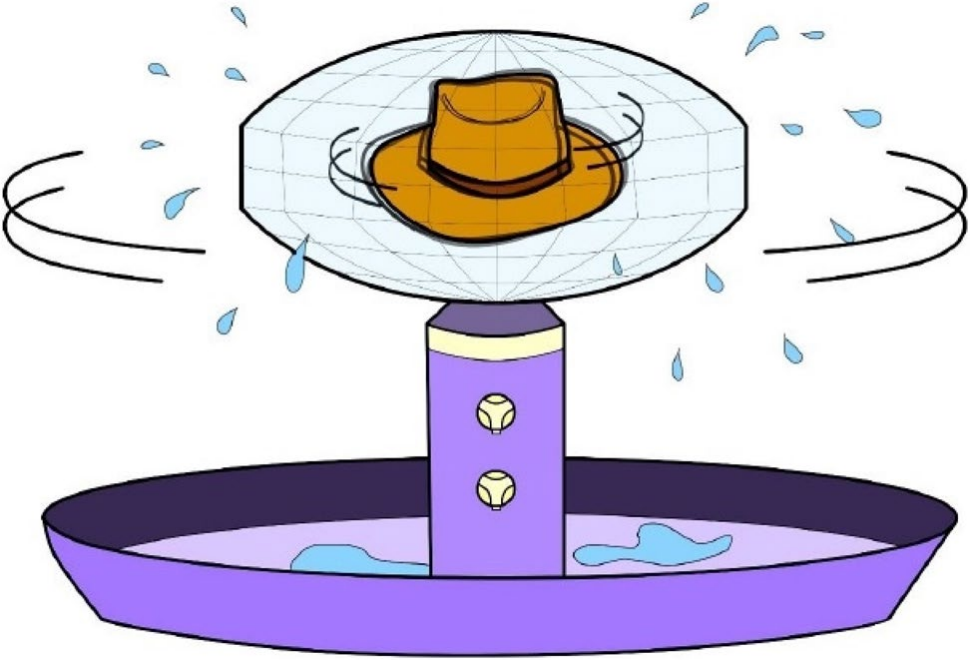
Example of a picture used during oral vocabulary training. A machine that is used to ‘vib’ soaking wet hats

This followed the procedure by Beyersmann, Wegener, et al. (2021). Participants were trained in small groups on one set of complex novel words (Set 1 or Set 2), with the other set constituting their untrained items. Sets were counterbalanced across groups. Participants were told that they would be learning about ‘Professor Parsnip’s Inventions’ and engaged in a range of activities to learn about the function and perceptual features of each invention. For example, they learned that “Professor Parsnip has invented a machine that vibs. The machine is used for drying hats. It is made of plastic and spins.” Each invention was paired with a picture demonstrating its features (Fig. 2). Eight items (four from each spelling predictability condition) were introduced and rehearsed twice on Day 1, the remaining eight were introduced and rehearsed twice on Day 2, and all 16 items were trained and rehearsed in the last session on Day 3. During rehearsal, participants were briefly reminded of each invention’s meaning and asked to repeat the associated novel word forms.

#### Picture-Naming (Posttraining Check)

To assess if participants had learned the spoken forms and their meanings, they were individually shown pictures of the inventions and accuracy was recorded for remembering novel words and their meaning. For example, participants were shown the picture of a machine used for drying hats (Fig. 2) and asked two questions: “What does this invention do?” and “Can you tell me what the invention is used for?” Children received two independent scores for correctly retrieving the novel word form (e.g., “It vibs.”), independently of their inflectional ending, and for correctly retrieving word meaning (e.g., “It is used to dry hats.”). In the case of an incorrect response, the experimenter provided the correct answer and participants were prompted to repeat it.

#### Eye-Tracking Experiment (First Orthographic Exposure)

In the eye-tracking experiment, participants encountered the novel stems for the first time, embedded in sentences. Word stems (*vib*) were embedded in a carrier sentence (e.g., *Sara put her soaking wet hat on the machine to vib it dry*). Carrier sentences were designed to be contextually rich, such that as participants read them, they would expect to see the word they had learned about during oral vocabulary training (Appendix B). All sentences appeared on a single line. Eye movements were monitored as sentences were read silently; 16 sentences contained reference to trained and 16 to untrained inventions. Half contained predictable (*vib*) and half unpredictable spellings (*bype*). Four filler sentences were included with novel inventions not learned by either group.

Eye movements were recorded using an EyeLink Portable Duo eye tracker (SR Research; Mississauga, Canada) in head-stabilized mode sampling at 2000 Hz as participants read sentences on a computer monitor. Each character subtended 0.3° of horizontal visual angle. Sentences were presented in black, Courier New font on a white background. Participants read binocularly, but only the movements of the right eye were monitored. A three-point initial calibration of the eye tracker was performed (maximum average error of 0.3), followed by three practice trials, and then the experimental sentences in four blocks of 10 with opportunities to rest in between. The experimenter triggered the beginning and end of each trial after the participants looked at a fixation cross to indicate their readiness. To promote attention to task, they were required to answer a (yes/no) question after each trial.

Eye movement dependent variables were extracted, capturing reading behaviour on the target word: first fixation duration (duration of initial fixation on the target); gaze duration (sum of all fixations made on the target before the eyes move past the target to a subsequent word within the sentence); total reading time (sum of all fixations on the target, including any regressions back to it); and regressions in (probability of making a regression back to the target from a later portion in the sentence).

#### Reading Aloud (Second Orthographic Exposure)

Participants read aloud all 16 trained and 16 untrained word stems, presented individually and in randomized order in the centre of a computer screen using DMDX software (Forster & Forster, 2003). Each trial consisted of an 800-ms fixation cross followed by the target, which remained until a response was given or until 4 seconds had elapsed. Participants were instructed to name each word as quickly and accurately as possible, while reaction times and response accuracy were assessed.

### Results

#### Assessment of Oral Vocabulary Learning: Picture Naming

Participants correctly recalled 7.47 of the 16 orally trained invention verbs (*SD* = 4.39). The difference in recall between participants who learned Set 1 (*M* = 6.43, *SD* = 4.61) and Set 2 (*M* = 8.64, *SD* = 3.79) was not significant, *t*(51) = 1.86, *p* = .07, nor was the difference in recall for items allocated predictable (*M* = 3.91, *SD* = 2.55) and unpredictable (*M* = 3.57, *SD* = 2.34) spellings, *t*(104) = 0.71, *p* = .48.

#### Eye movements

Data were analyzed in the R computing environment (R Core Team, 2021). Linear mixed-effects models were constructed using the *lme4* package (Bates et al., 2020) and *p* values were obtained using the *lmer Test* package (Kuznetsova et al., 2017). The area of interest was the invention name, hereafter referred to as the target word. Fixations shorter than 80 milliseconds and within half a degree of the previous or next fixation were merged, and any remaining fixations shorter than 80 milliseconds or longer than 1,200 milliseconds were deleted. Trials were removed if a blink occurred on the target word during first pass, or if any of the three prespecified interest areas—target word, pretarget text, posttarget text—were not fixated. Following these cleaning steps, 86.32% of the experimental data remained.

**Fig. 3:**
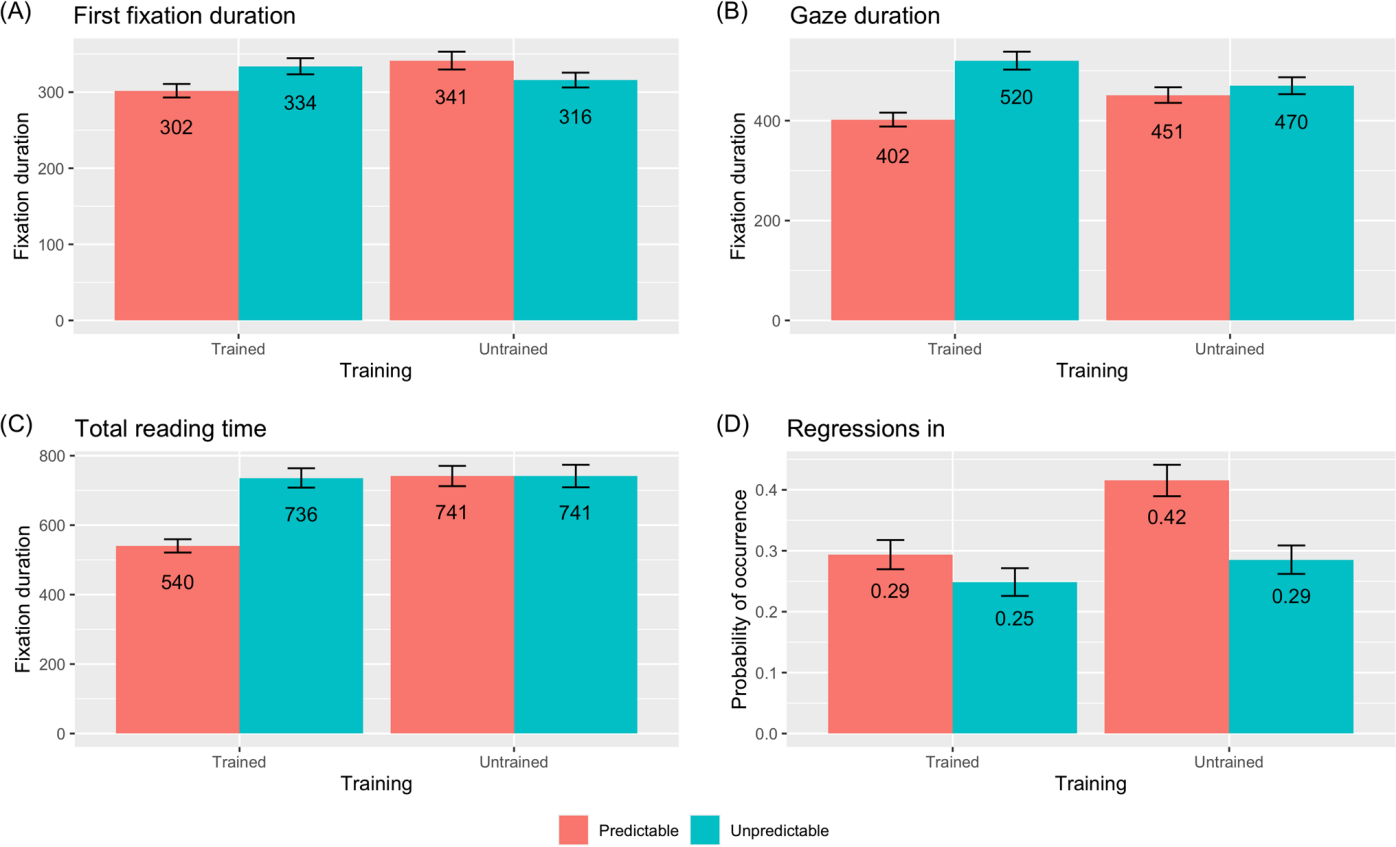
Arithmetic means and standard errors of target word fixation durations and probability of rereading. First fixation duration, gaze duration, and total reading time are expressed in milliseconds while regressions in reflects likelihood of occurrence

Models were run for each of the dependent variables of interest: first fixation duration, gaze duration, total reading time, and regressions in. Reading-time data were log transformed prior to analysis. Fixed effects were training, spelling predictability, and their interaction, and these were deviation coded (−0.5, +0.5). Participants and items were entered as random effects. As described in the preregistration, a data-driven approach to model selection was employed. As per Barr and colleagues (2013), models were computed with the maximal random effects structure, but these were overfitted (Baayen, 2008). Next, the random intercepts model was computed and random slopes were added incrementally. The highest converging nonsingular models are reported. Interactions were unpacked using the *phia* package (Rosario-Martino & Fox, 2015). For ease of interpretation, arithmetic means and standard errors for each of the dependent variables appear in Fig. 3.

Model outputs are presented in Table 2. For *first fixation duration*, the fixed effects of training, spelling predictability, and their interaction all failed to reach statistical significance. For *gaze duration*, the fixed effect of training was not significant. There was a fixed effect of spelling predictability such that predictable items received shorter fixations than unpredictable items, and there was a significant two-way interaction between training and spelling predictability. Interaction contrasts showed that there was an effect of spelling predictability for trained items (χ^2^ *=* 16.53, *p* < .001) but not for untrained items (χ^2^ = 0.12, *p* = .730). Further, there was no effect of training either for predictable (χ^2^ = 2.56, *p* = .110), nor for unpredictable items (χ^2^ = 3.86, *p* = .099). For *total reading time*, neither training nor spelling predictability were significant. However, there was a significant two-way interaction between training and spelling predictability. Interaction contrasts showed that there was an effect of spelling predictability for trained items (χ^2^ = 11.27, *p* = .002) but not for untrained items (χ^2^ = 0.48, *p* = .491). Additionally, there was also an effect of training for predictable (χ^2^ = 10.94, *p* = .002), but not for unpredictable items (χ^2^ = 0.62, *p* = .441). For *regressions in*, there was a fixed effect of training such that trained items were less likely to receive return fixations than untrained items. There was also an effect of spelling predictability such that predictable items were more likely to receive return fixations than unpredictable items. The interaction between training and spelling predictability was not signifcant.

**Table 2:** Linear mixed-effects models for the eye-movement data

		Training	Predictability	Interaction
First fixation duration				
	*b*	0.01	0.01	−1.12
	*SE*	0.03	0.03	0.06
	*t*	0.35	0.18	−1.94
	*p*	.733	.858	.063
Gaze duration				
	*b*	−0.01	0.12	−0.19
	*SE*	0.04	0.05	0.08
	*t*	−0.22	2.32	−2.517
	*p*	.827	.0269	.0181
Total reading time				
	*b*	0.11	0.09	−0.35
	*SE*	0.06	0.08	0.12
	*t*	1.76	1.173	−2.89
	*p*	.089	.249	.007
Regressions in				
	*b*	0.36	−0.50	−0.45
	*SE*	0.15	0.15	0.28
	*z*	2.33	−2.97	−1.62
	*p*	.020	.003	.106

### Reading Aloud

**Fig. 4:**
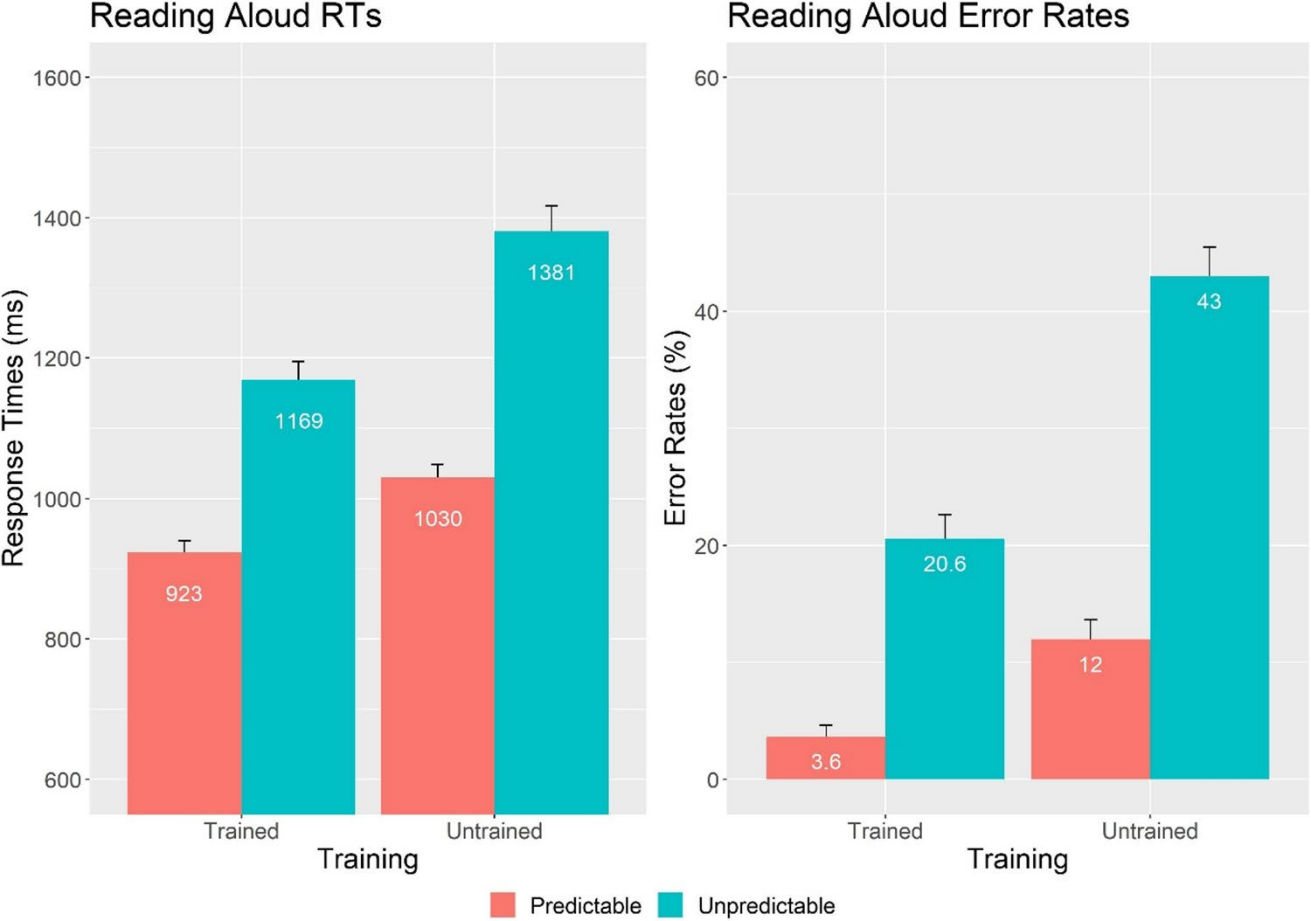
Mean response times (in milliseconds; left panel), error rates (in %; right panel), and standard errors of the reading-aloud task

Five participants were excluded, because error rates were above 50%. Incorrect responses were removed from the RT analysis (19.8% of all data). We used linear mixed-effect modelling to perform the main analyses, including factors spelling predictability (predictable, unpredictable), training (trained, untrained), and the interaction between spelling predictability and training. The random effects structure was selected following the same procedure as in the eye-tracking analyses, choosing the highest converging nonsingular model. Response times were logarithmically transformed. The model was refitted after excluding data points whose standardized residuals were larger than 2.5 in absolute value (2.1% of the data; Baayen, 2008). RT analyses revealed a significant effect of spelling predictability, χ^2^(1) = 54.14, *p* < .001, showing that predictable novel words were read faster than unpredictable novel words, and a significant effect of training, χ^2^(1) = 64.35, *p* < .001, indicating that trained novel words were read faster than untrained novel words (Fig. 4). There was also a significant interaction between spelling predictability and training, χ^2^(1) = 3.98, *p* = .046, which went in the opposite direction of the interaction seen in the eye-tracking data, showing that the spelling predictability effect was reduced for trained, *χ*^*2*^*(*1) *=* 38.62, *p* < .001, compared with untrained items, χ^2^(1) *=* 50.93, *p* < .001.

Error rates were analyzed by applying a binomial variance assumption to the trial-level binary data using the function *glmer* as part of the R package *lme4*. There was a significant effect of spelling predictability, χ^2^(1) = 45.15, *p* < .001, showing that participants made fewer errors reading predictable than unpredictable novel words, and a significant effect of training, χ^2^(1) = 56.69, *p* < .001, indicating that participants made fewer errors reading trained than untrained novel words. The interaction between spelling predictability and training was not significant, χ^2^(1) = 0.02, *p* = .892.

## Discussion

The present study used a novel word-training paradigm to examine the mechanisms by which children integrate oral vocabulary knowledge into their reading. On three consecutive days, children were taught the oral forms of novel morphologically complex words (e.g., /vɪbɪŋ/, /vɪbd/, /vɪbz/) consisting of a novel word stem (*vib*) and an existing inflectional affix (“-s”, “-ing”, or “-ed”). Half of the novel stems had a predictable spelling (*vib*) and half an unpredictable spelling (*bype*). Following training, participants then read novel word stems, half trained and half untrained, for the first time, while their eye-movements were monitored. In addition, a reading-aloud task was administered where children were exposed to the written novel word forms for a second time.

The eye-tracking results revealed two key findings that were in line with the study’s preregistered hypotheses (https://aspredicted.org/3vs5s.pdf). First, there was a significant two-way interaction between training and spelling predictability on gaze duration, suggesting that trained stems with predictable spellings attracted shorter looking times than trained stems with unpredictable spellings, whereas no such difference was observed in the untrained control condition. This result provides a window into the early, automatic stages of reading when children were exposed to the printed form of novel stems for the very first time and suggests that individuals who are still in the process of learning to read are already able to make orthographic predictions of embedded novel stems from spoken input. Second, a significant two-way interaction between training and spelling predictability was also observed on total reading time. This result thus confirms the robustness of the orthographic skeleton effect in children, showing that trained stems with predictable spelling continued to be fixated for shorter periods of time than trained stems with unpredictable spellings throughout the later stages of reading. A likely interpretation of the present data is that orthographic expectancies were formed automatically without any explicit control of the applied mappings (e.g., Huettig et al., 2022), as previously evidenced by studies reporting orthographic influences on spoken-word processing within auditory lexical decision (e.g., Chéreau et al., 2007; Pattamadilok et al., 2007; Perre et al., 2009; Taft et al., 2008; Ziegler et al., 2004; Ziegler et al., 2008). It is also possible that participants actively and strategically used their knowledge of phoneme-to-grapheme mappings as a mnemonic memory aid during learning of the oral vocabulary, but the current study did not test this possibility.

The present findings not only suggest that children’s ability to map phonemes to graphemes is already well developed enough to support the generation of orthographic skeletons, but also point to the important role of morphological knowledge within the process of building orthographic expectancies during oral word training (e.g., Beyersmann et al., 2012; Beyersmann, Mousikou, et al., 2021; Quémart et al., 2011). Our data show that children’s ability to predict orthographic forms based on spoken forms is not only limited to morphologically simple words (Wegener et al., 2018; Wegener et al., 2020) but is also evident for novel stems embedded in morphologically complex words. There are several possibilities as to how participants extracted the embedded morphemic content during spoken-word training. One possibility is that children did not only acquire the spoken complex novel words holistically but simultaneously learned to identify the spoken forms of the embedded morphemic constituents (e.g., /vɪbz/, /vɪb/, and /z/), prior to generating orthographic skeletons during spoken-word training (*vib*). Another possibility is that the morphologically complex form was first mapped onto an orthographic whole-word form (*vibs*), which then in turn activated the morphemic subunits (*vib* + *s*). Whichever was the case, this kind of sensitivity to the morphological structure of complex words is consistent with decompositional as well as distributional theories of morphological processing (for a recent review, see Stevens & Plaut, 2022). The decompositional view (e.g., Beyersmann, Bolger, et al., 2019; Grainger & Beyersmann, 2017; Rastle et al., 2004; Taft, 2003) would predict that children segmented the complex novel words into stem and suffix which then allowed them to form an orthographic prediction of the embedded stem constituent. The distributional view would assume that the morphological regularity of the spoken input signal is captured in the links between phonology, orthography, and semantics (e.g., Plaut & Gonnerman, 2000; Rueckl & Raveh, 1999; Seidenberg & Plaut, 2014) and therefore equally offers an explanation for the embedded morpheme effects.

Although the present study cannot clearly adjudicate between these different possibilities, it is likely that children were able to draw on their morphological knowledge during spoken-word training and extract the oral form of the novel stem prior to generating an orthographic expectation. Primary school children already have advanced spoken language by the time they begin to learn to read and are proficient at identifying morphological structures in oral language from preschool age (e.g., Carlisle, 2000; Deacon et al., 2012; Deacon & Bryant, 2006). In contrast, research examining children’s ability to identify morphological structures from print shows that the acquisition of a reading mechanism by which written words are segmented into morphemic subunits develops comparatively late (e.g., Beyersmann et al., 2012; Dawson et al., 2018, 2021; Schiff et al., 2012). One could therefore assume that the observed effect of embedded word acquisition is more likely due to the children’s profound knowledge of spoken (rather than written) complex words and their ability to segment the spoken input signal into morphemic subunits before mapping them onto the corresponding orthographic forms, a point that will require further exploration in the future.

A third key finding was the significant training by spelling predictability interaction in the response times of the reading-aloud task, where participants were exposed to the orthographic form of the novel stems for a second time. Here, the interaction went in the opposite direction to the eye-tracking data, showing a smaller predictability effect for trained than untrained items. These results shed further light onto the nature of the orthographic skeleton effect, suggesting that the orthographic expectations that children formed during oral word training were updated during the first orthographic exposure. This finding coincides with previous work (Beyersmann, Wegener, et al., 2021; Wegener et al., 2020), suggesting that readers rapidly correct the orthographic expectations that are formed during oral word training (/baɪp/–*bipe*) once an alternate orthographic form is encountered in print (*bype*). This in turn predicts that if the outcome measures were administered in the reverse order (i.e., reading aloud preceding eye tracking), we would expect to observe key evidence for the orthographic skeleton effect on the task administered first (reading aloud). This is because influences arising from phonology-to-orthography should be most apparent at the earliest point in learning (e.g., McKague et al., 2008; Wegener et al., 2020). Wegener et al. (2020) used eye tracking to test three consecutive orthographic exposures and showed that orthographic expectancies were updated between the second and third exposures. This supports the idea that readers gradually correct their expectancies when the same outcome metric is used. Hence, the current data not only suggest that the formation of orthographic skeletons represents an important milestone in the beginning stages of children’s reading acquisition but also showcases the flexibility by which children use their reading experience to update their knowledge of orthographic word forms.

A question for future research may be how soon during reading development children begin to benefit from the formation of orthographic skeletons in their reading. The evidence presented here was derived from Australian primary school children including Grades 3–5, where even the youngest participants (i.e., third graders) had already experienced three consecutive years of formal reading instruction (for related evidence from fourth graders, see also Wegener et al., 2018; Wegener et al., 2020). It is less clear if younger children with less well-developed orthographic knowledge would demonstrate the same kind of ability in predicting orthographic forms from spoken-word exposure. Thus, questions remain regarding the early developmental trajectory of the orthographic skeleton effect, which may be addressed by extending the current work to even younger, less fluent readers.

Another prospect for future work relates to the question of whether the acquisition of embedded word skeletons is limited to embedded words occurring in a genuine morphological context (e.g., “neshed”) or also applies in situations where words are embedded in nonaffixed novel words (e.g., “neshel”). Although the current data clearly suggest that orthographic expectations of embedded stems were formed during oral word training, this does not rule out that orthographic expectations may also be formed for words embedded in morphologically simple novel words. Indeed, embedded word activations are predicted by the vast majority of decompositional as well as distributional theories of morphological processing (e.g., Stevens & Plaut, 2022). In particular, the word and affix model (Beyersmann & Grainger, 2022) supposes that the activation of embedded stems is handled by an entirely nonmorphological process of edge-aligned embedded word activation, therefore allowing for the activation of words embedded in morphologically simple words (e.g., *far* in *farm*). Evidence from orthographic learning shows that children’s orthographic learning of novel words facilitated processing of novel items that appeared to be morphologically related as well as those that only shared an orthographic relationship (Tucker et al., 2016). However, it is further predicted (e.g., Beyersmann & Grainger, 2022; Grainger & Beyersmann, 2017) that lateral inhibition between simultaneously active, morphologically unrelated, lexical candidates (e.g., *far* and *farm*) will typically lead to lower activation thresholds of the shorter, embedded item (e.g., Beyersmann, Grainger, et al., 2019). While such lateral activation also challenges the activation of words embedded in morphologically complex words (e.g., *farm* in *farmer*), this does not typically lead to a decrease in the activation of the embedded item, given its semantic and morphological overlap with the whole word representation. An informative follow-up of our present results would therefore be a study examining novel words consisting of combinations of stems and nonmorphemic endings, to more precisely determine the kind of mechanisms that are used to extract the stem during oral vocabulary exposure.

In sum, the present training study sheds light on the mechanisms by which children acquire novel words and demonstrates the prominent role of prior spoken-language knowledge during reading development. The combined eye-tracking and reading-aloud data suggest that the acquisition of written words already begins before the orthographic forms are encountered in print. Children were able to extract and form orthographic expectancies of novel word stems, suggesting that orthographic expectancies involve setting up a complex set of orthographic predictions for not just whole words but also stems embedded in words with multiple morphemes. The current study highlights the robustness of the orthographic skeleton effect in developing readers and shows that children who are still in the process of learning to read are already expert at integrating spoken-word knowledge into their reading processes in highly automatic and flexible ways.

## Appendix A

**Table:**

	Set 1		Set 2	
	complex words	stem morphemes	complex words	stem morphemes
Predictable	/dʒevɪŋ/ /dʒevd/ /dʒevz/	jev	/temɪŋ/ /temd/ /temz/	tem
	/jægɪŋ/ /jægd/ /jægz/	yag	/nɪdɪŋ/ /nɪdəd/ /nɪdz/	nid
	/vɪbɪŋ/ /vɪbd/ /vɪbz/	vib	/dʒɪtɪŋ/ /dʒɪtɪd/ /dʒɪts/	jit
	/tʌpɪŋ/ /tʌpt/ /tʌps/	tup	/jæbɪŋ/ /jæbd/ /jæbz/	yab
	/ne∫ɪŋ/ /ne∫t/ /ne∫ɪz/	nesh	/vɪ∫ɪŋ/ /vɪ∫t/ /vɪ∫ɪz/	vish
	/tʃɒbɪŋ/ /tʃɒbd/ /tʃɒbz/	chob	/∫epɪŋ/ /∫ept/ /∫eps/	shep
	/∫ʌgɪŋ/ /∫ʌgd/ /∫ʌgz/	shug	/θɒgɪŋ/ /θɒgd/ /θɒgz/	thog
	/θʌbɪŋ/ /θʌbd/ /θʌbz/	thub	/tʃɪgɪŋ/ /tʃɪgd/ /tʃɪgz/	chig
Unpredictable	/viːmɪŋ/ /vɪːmd/ /vɪːmz/	veme	/juːnɪŋ/ /juːnd/ /juːnz/	yune
	/baɪpɪŋ/ /baɪpt/ /baɪps/	bype	/kaɪvɪŋ/ /kaɪvd/ /kaɪvz/	kyve
	/jɜːpɪŋ/ /jɜːpt/ /jɜːps/	yirp	/bɜːvɪŋ/ /bɜːvd/ /bɜːvz/	birv
	/kɔɪbɪŋ/ /kɔɪbd/ /kɔɪbz/	koyb	/dʒaɪfɪŋ/ /dʒaɪft/ /dʒaɪfs/	jayf
	/dʒiːbɪŋ/ /dʒiːbd/ /dʒiːbz/	jeabb	/miːfɪŋ/ /miːft/ /miːfs/	meaph
	/fɜːfɪŋ/ /fɜːft/ /fɜːfs/	phirf	/gʌzIŋ/ /gʌzd/ /gʌzɪz/	ghuzz
	/gækɪŋ/ /gækt/ /gæks/	ghakk	/fegɪŋ/ /fegd/ /fegz/	phegg
	/mɜːbɪŋ/ /mɜːbd/ /mɜːbz/	mirbe	/veɪpɪŋ/ /veɪpt/ /veɪps/	vaype

## Appendix B

**Table:**

Set 1	
1	Rick put his dirty socks into the machine to jev them clean.
2	Diana put the best orange on the machine to veme the juice.
3	Pam put the dirty flowers under the machine to yag them shiny.
4	Max put his food into the machine to bype the yucky peas.
5	Sara put her soaking wet hat on the machine to vib it dry.
6	Lucy loaded all the rubbish into the machine to yirp it for recycling.
7	Lucas put his sore tummy beside the machine to tup it better again.
8	Jennifer put all her soggy chips under the machine to koyb them crispy.
9	Nick put the playing cards into the machine to nesh before starting the game.
10	Rex put the tennis balls into the machine to jeabb as he played fetch.
11	James put the picture of the girl into the machine to chob her name.
12	Jane put her cold and sore feet into the machine to phirf them warm.
13	Matt put his feet into the machine to shug quickly up the wall.
14	Sam saw a black bird and then made the machine ghakk to hear it sing.
15	Ben put the machine into the fish tank to thub the glass clean again.
16	Pip waited for the brushes on the machine to mirbe the sand from his body.
Set 2	
1	Rick put his dirty socks into the machine to tem them clean.
2	Diana put the best orange on the machine to yune the juice.
3	Pam put the dirty flowers under the machine to nid them shiny.
4	Max put his food into the machine to kyve the yucky peas.
5	Sara put her soaking wet hat on the machine to jit it dry.
6	Lucy loaded all the rubbish into the machine to birv it for recycling.
7	Lucas put his sore tummy beside the machine to yab it better again.
8	Jennifer put all her soggy chips under the machine to jayf them crispy.
9	Nick put the playing cards into the machine to vish before starting the game.
10	Rex put the tennis balls into the machine to meaph as he played fetch.
11	James put the picture of the girl into the machine to shep her name.
12	Jane put her cold and sore feet into the machine to ghuzz them warm.
13	Matt put his feet into the machine to thog quickly up the wall.
14	Sam saw a black bird and then made the machine phegg to hear it sing.
15	Ben put the machine into the fish tank to chig the glass clean again.
16	Pip waited for the brushes on the machine to vaype the sand from his body.

## References

[CR1] Baayen RH (2008). *Analyzing linguistic data: A practical introduction to statistics using R*.

[CR2] Barr, D. J., Levy, R., Scheepers, C., & Tily, H. J. (2013). Random effects structure for confirmatory hypothesis testing: Keep it maximal. *Journal of Memory & Language, 68*(3). 10.1016/j.jml.2012.11.001 10.1016/j.jml.2012.11.001 PMC3881361 24403724

[CR3] Bates, D., Maechler, M., Bolker, B., & Walker, S. (2020). *Package ‘lme4’*. https://cran.r-project.org/web/packages/lme4/lme4.pdf

[CR4] Berko J (1958). The child’s learning of English morphology. Word.

[CR5] Beyersmann E, Bolger D, Pattamadilok C, New B, Ziegler JC, Grainger J (2019). Morphological processing without semantics: An ERP study with spoken words. Cortex.

[CR6] Beyersmann E, Castles A, Coltheart M (2012). Morphological processing during visual word recognition in developing readers: Evidence from masked priming. The Quarterly Journal of Experimental Psychology.

[CR7] Beyersmann E, Grainger J, Crepaldi D (2022). The role of embedded words and morphemes in reading. *Current issues in the psychology of language*.

[CR8] Beyersmann E, Grainger J, Taft M (2019). *Evidence for embedded word length effects in complex nonwords*.

[CR9] Beyersmann E, Mousikou P, Schroeder S, Javourey-Drevet L, Ziegler JC, Grainger J (2021). The dynamics of morphological processing in developing readers: A cross-linguistic masked priming study. Journal of Experimental Child Psychology.

[CR10] Beyersmann E, Wegener S, Nation K, Prokupzcuk A, Wang H-C, Castles A (2021). Learning morphologically complex spoken words: Orthographic expectations of embedded stems are formed prior to print exposure. Journal of Experimental Psychology: Learning, Memory and Cognition.

[CR11] Bryant P, Nunes T (2008). Morphemes, spelling and develpment: Comments on “the timing and mechanisms of children's use of morphological information in spelling” by S. Pacton and H. Deacon. Cognitive Development.

[CR12] Carlisle JF (2000). Awareness of the structure and meaning of morphologically complex words: Impact on reading. Reading and Writing: An Interdisciplinary Journal.

[CR13] Castles A, Coltheart M, Larsen L, Jones P, Saunders S, McArthur G (2009). Assessing the basic components of reading: A revision of the Castles and Coltheart test with new norms. Australian Journal of Learning Difficulties.

[CR14] Chéreau C, Gaskell MG, Dumay N (2007). Reading spoken words: Orthographic effects in auditory priming. Cognition.

[CR15] Dawson N, Rastle K, Ricketts J (2018). Morphological effects in visual word recognition: Children, adolecents, and adults. Journal of Experimental Psychology: Learning, Memory and Cognition.

[CR16] Dawson N, Rastle K, Ricketts J (2021). Finding the man amongst many: A developmental perspective on mechanisms of morphological decomposition. Cognition.

[CR17] Deacon SH, Benere J, Pasquerella A (2012). Reciprocal relationship: Children’s morphological awareness and their reading accuracy across grades 2 to 3. Developmental Psychology.

[CR18] Deacon SH, Bryant P (2006). This turnip’s not for turning: Children’s morphological awareness and their use of root morphemes in spelling. British Journal of Developmental Psychology.

[CR19] Deacon SH, Kirby JR (2004). Morphological awareness: Just “more phonological”? The roles of morphological and phonological awareness in reading development. Applied PsychoLinguistics.

[CR20] Duff FJ, Reen G, Plunkett K, Nation K (2015). Do infant vocabulary skills predict school-age language and literacy outcomes?. Journal of Child Psychology and Psychiatry.

[CR21] Forster KI, Forster JC (2003). DMDX: A windows display program with millisecond accuracy. Behavior Research Methods, Instruments, & Computers.

[CR22] Grainger, J., & Beyersmann, E. (2017). Edge-aligned embedded word activation initiates morpho-orthographic segmentation. In B. H. Ross (Ed.), *The psychology of learning and motivation* (Vol. 67, pp. 285–317). Elsevier Academic Press.

[CR23] Huettig, F., Audring, J., & Jackendoff, R. (2022). A parallel architecture perspective on pre-activation and prediction in language processing. *Cognition, 105050*(224). 10.1016/j.cognition.2022.105050 10.1016/j.cognition.2022.105050 35398592

[CR24] Jevtovic M, Antzaka A, Martin CD (2022). Gepo with a G, or Jepo with a J? Skilled readers generate orthographic expectations for novel spoken words even when spelling is uncertain. Cognitive Science.

[CR25] Johnston MB, McKague M, Pratt C (2004). Evidence for an automatic orthographic code in the processing of visually novel word forms. Language and Cognitive Processes.

[CR26] Kuznetsova, A., Brockhoff, P. B., & Christensen, R. H. B. (2017). lmerTest package: Tests in linear mixed effects models. *Journal of Statistical Software, 82*(13), 1–26. 10.18637/jss.v082.i13

[CR27] Lee J (2011). Size matters: Early vocabulary as a predictor of language and literacy competence. Applied PsychoLinguistics.

[CR28] McKague M, Davis C, Pratt C, Johnston MB (2008). The role of feedback from phonology to orthography in orthographic learning: An extension of item-based accounts. Journal of Research in Reading.

[CR29] McKague M, Pratt C, Johnston MB (2001). The effect of oral vocabulary on reading visually novel words: A comparison of the dual-route-cascaded and triangle frameworks. Cognition.

[CR30] Nation K, Cocksey J (2009). Beginning readers activate semantics from sub-word orthography. Cognition.

[CR31] Nation K, Snowling MJ (2004). Beyond phonological skills: Broader language skills contribute to the development of reading. Journal of Research in Reading.

[CR32] Pattamadilok C, Morais J, Ventura P, Kolinsky R (2007). The locus of the orthographic consistency effect in auditory word recognition: Further evidence from French. Language and Cognitive Processes.

[CR33] Perre L, Pattamadilok C, Montant M, Ziegler JC (2009). Orthographic effects in spoken language: On-line activation or phonological restructuring?. Brain Research.

[CR34] Plaut DC, Gonnerman LM (2000). Are non-semantic morphological effects incompatible with a distributed connectionist approach to lexical processing?. Language and Cognitive Processes.

[CR35] Quémart P, Casalis S, Colé P (2011). The role of form and meaning in the processing of written morphology: A priming study in French developing readers. Journal of Experimental Child Psychology.

[CR36] R Core Team. (2021). *R: A language and environment for statistical computing*. R Foundation for Statistical Computing. https://www.R-project.org/

[CR37] Rastle, K. (2018). The place of morphology in learning to read in English. *Cortex.*10.1016/j.cortex.2018.02.008 10.1016/j.cortex.2018.02.008 29605387

[CR38] Rastle K, Davis MH, New B (2004). The broth in my brother's brothel: Morpho-orthographic segmentation in visual word recognition. Psychonomic Bulletin & Review.

[CR39] Rosario-Martino, H., & Fox, J. (2015). *Package ‘phia’*.

[CR40] Rueckl JG, Raveh M (1999). The influence of morphological regularities on the dynamics of a connectionist network. Brain and Language.

[CR41] Schiff R, Raveh M, Fighel A (2012). The development of the Hebrew mental lexicon: When morphological representations become devoid of their meaning. Scientific Studies of Reading.

[CR42] Seidenberg MS, Plaut DC (2014). Quasiregularity and its discontents: The legacy of the past tense debate. Cognitive Science.

[CR43] Stevens, P., & Plaut, D. C. (2022). From decomposition to distributed theories of morphological processing in reading. *Psychonomic Bulletin & Review*. 10.3758/s13423-022-02086-0 10.3758/s13423-022-02086-0 35595965

[CR44] Taft M, Assink E, Sandra D (2003). Morphological representation as a correlation between form and meaning. *Reading complex words*.

[CR45] Taft M, Castles A, Davis C, Lazendic G, Nguyen-Hoan M (2008). Automatic activation of orthography in spoken word recognition: Pseudohomograph priming. Journal of Memory and Language.

[CR46] Tucker R, Castles A, Laroche A, Deacon SH (2016). The nature of orthographic learning in self-teaching: Testing the extent of transfer. Journal of Experimental Child Psychology.

[CR47] Wegener S, Wang HC, de Lissa P, Robidoux S, Nation K, Castles A (2018). Children reading spoken words: Interactions between vocabulary and orthographic expectancy. Developmental Science.

[CR48] Wegener S, Wang HC, Nation K, Castles A (2020). Tracking the evolution of orthographic expectancies over building visual experience. Journal of Experimental Child Psychology.

[CR49] Ziegler JC, Ferrand L, Montant M (2004). Visual phonology: The effects of orthographic consistency on different auditory word recognition tasks. Memory & Cognition.

[CR50] Ziegler JC, Petrova A, Ferrand L (2008). Feedback consistency effects in visual and auditory word recognition: Where do we stand after more than a decade?. Journal of Experimental Psychology: Learning, Memory, and Cognition.

